# SERR Spectroelectrochemical Study of Cytochrome *cd*_1_ Nitrite Reductase Co-Immobilized with Physiological Redox Partner Cytochrome *c*_552_ on Biocompatible Metal Electrodes

**DOI:** 10.1371/journal.pone.0129940

**Published:** 2015-06-19

**Authors:** Célia M. Silveira, Pedro O. Quintas, Isabel Moura, José J. G. Moura, Peter Hildebrandt, M. Gabriela Almeida, Smilja Todorovic

**Affiliations:** 1 Instituto de Tecnologia Química e Biológica, Universidade Nova de Lisboa, Oeiras, Portugal; 2 UCIBIO, REQUIMTE, Departamento de Química, Faculdade de Ciências e Tecnologia, Universidade Nova de Lisboa, Caparica, Portugal; 3 Technische Universitat Berlin, Institut fur Chemie, Berlin, Germany; 4 Centro de Investigação Interdisciplinar Egas Moniz (CiiEM), Instituto Superior de Ciências da Saúde Egas Moniz, Caparica, Portugal; US Naval Reseach Laboratory, UNITED STATES

## Abstract

Cytochrome cd1 nitrite reductases (*cd*_1_NiRs) catalyze the one-electron reduction of nitrite to nitric oxide. Due to their catalytic reaction, *cd*_1_NiRs are regarded as promising components for biosensing, bioremediation and biotechnological applications. Motivated by earlier findings that catalytic activity of *cd*_1_NiR from *Marinobacter hydrocarbonoclasticus* (*Mhcd*_1_) depends on the presence of its physiological redox partner, cytochrome *c*_552_ (cyt *c*_552_), we show here a detailed surface enhanced resonance Raman characterization of *Mhcd*_1_ and cyt *c*_552_ attached to biocompatible electrodes in conditions which allow direct electron transfer between the conducting support and immobilized proteins. *Mhcd*_1_ and cyt *c*552 are co-immobilized on silver electrodes coated with self-assembled monolayers (SAMs) and the electrocatalytic activity of Ag // SAM // *Mhcd*_1_ // cyt *c*_552_ and Ag // SAM // cyt *c*_552_ // *Mhcd*_1_ constructs is tested in the presence of nitrite. Simultaneous evaluation of structural and thermodynamic properties of the immobilized proteins reveals that cyt *c*_552_ retains its native properties, while the redox potential of apparently intact *Mhcd*_1_ undergoes a ~150 mV negative shift upon adsorption. Neither of the immobilization strategies results in an active *Mhcd*_1_, reinforcing the idea that subtle and very specific interactions between *Mhcd*_1_ and cyt *c*_552_ govern efficient intermolecular electron transfer and catalytic activity of *Mhcd*_1_.

## Introduction

Cytochrome *cd*_1_ nitrite reductases (*cd*_1_NiRs) are periplasmic proteins involved in the second step of the denitrification pathway (NO_3_^-^→***NO***_***2***_^***-***^ ***→NO***→N_2_O→N_2_), corresponding to the reduction of nitrite to nitric oxide [1–3]. *cd*_1_NiRs are homodimeric proteins containing one *c*-type and one *d*_1_-type heme per subunit. The heme *c* is thought to be the electron entry site, receiving electrons from small electron donor proteins, such as *c*-type cytochromes (cytochrome *c*_552_ [cyt *c*_552_], cytochrome *c*_551_, cytochrome *c*_550_) or copper proteins (azurin, pseudoazurin); the electrons are then used for nitrite reduction by heme *d*_1_ [1–3]. This cofactor is quite distinct from other types of hemes, due to its asymmetric porphyrin ring and a highly ruffled structure. So far heme *d*_1_ has only been identified in *cd*_1_NiR enzymes isolated from denitrifying bacterial species, e.g. *Marinobacter hydrocarbonoclasticus*, *Pseudomonas stutzeri*, *Pseudomonas aeruginosa* and *Paracoccus pantotrophus* [1,2,4]. The enzymes from the last two organisms have been thoroughly characterized, mainly by X-ray crystallography and fast kinetics. Unusual structural and catalytic features have been reported concerning, in particular, i) activation mechanisms, which involve redox driven structural changes, including conformational rearrangements and heme ligand exchange [5–7], and ii) the release of the reaction product NO from the active site, to avoid the formation of a dead-end product (i.e. Fe^2+^-NO), since NO has a high affinity towards ferrous hemes [8–11]. Crystallographic structures of *P*. *pantotrophus* (*Ppcd*_1_) and the *P*. *aeruginosa* (*Pacd*_1_) *cd*_1_NiRs reveal different cofactor coordination patterns in the oxidized states and similar catalytically competent reduced forms [6,12]; the information about structural and mechanistic properties of *cd*_1_NiR from *M*. *hydrocarbonoclasticus* (*Mhcd*_1_) is lagging behind.

Due to the reaction that they catalyze, *cd*_1_NiRs are considered to be promising biocatalysts for the construction of electrochemical nitrite biosensors. These devices are expected to be able to selectively quantify nitrite in complex matrices and have broad applications, e.g. in drinking water regulation, environmental monitoring, clinical diagnosis and biomedical research [13,14]. A widely used approach for fabricating 3^rd^ generation biosensors consists of the immobilization of the enzyme on an electrode that serves as a controllable electron source to drive the reaction cycle. The communication between the enzyme and the electrode relies on efficient direct electron transfer (ET), thereby increasing selectivity, simplifying the manufacturing process and reducing the number of components of the device. One of the major obstacles in the development of these devices is the immobilization of the enzyme in the native state, while maintaining good electrical contact with the transducer surface and ensuring high catalytic efficiency [15,16]. The electrochemical methods, which are typically used to monitor the performance of a biosensor, cannot provide information on the molecular origin of altered (or absent) catalytic or redox activity of the enzyme, which is often a consequence of immobilization induced structural changes. This can be overcome by coupling the electrochemical with spectroscopic methods, which give insights into the structural features of immobilized proteins [17,18]. In the case of *cd*_1_NiRs, information on structural features of cofactors can be provided by resonance Raman (RR) spectroscopy that selectively probes redox, coordination and spin states of the heme groups upon excitation in resonance with their electronic transitions. When the protein is in close proximity to a nanostructured Ag surface, both plasmonic and resonance enhancements are matched using 413 nm excitation. Then the RR bands become further enhanced by several orders of magnitude (surface enhanced RR, SERR) allowing to probe the catalytic and/or redox site of the immobilized proteins only [19–21]. Moreover, a comparison of RR spectra in solution with SERR spectra of the adsorbed protein unambiguously reveals immobilization induced conformational alterations, if present.

Previous electrochemical studies of *cd*_1_NiR showed that the enzyme is capable of nitrite reduction only in the presence of putative electron donor proteins. Using non-physiological redox mediators (e.g. yeast cyt *c* or ferricyanide) results in only residual electrocatalytic response [22,23]. Enzymatic activity could be measured with *cd*_1_NiR and its physiological electron donor in solution, incorporated into polymeric films or entrapped with a dialysis membrane on the electrode surface [22–24]. However, despite all the efforts, including our own work, up to date there has been no report of direct ET between *cd*_1_NiRs and electrode surfaces [22,23,25].

To further explore the potential use of *Mhcd*_1_ for the development of nitrite biosensors, in this work we have searched for conditions which lead to a functional catalytic complex between *Mhcd*_1_ and its physiological electron donor, cyt *c*_552_. Direct contact of the proteins with the electrode surface, which can cause protein denaturation, was avoided by functionalization of the metal electrodes with alkanethiol based self-assembled monolayers (SAMs). SERR spectroelectrochemistry was employed to individually characterize *Mhcd*_1_, cyt *c*_552_ and their complexes adsorbed on biocompatible metal electrodes and evaluate the impact of immobilization on the structural and thermodynamic properties of the proteins. Cyclic voltammetry was used to probe the catalytic activity of the immobilized *Mhcd*_1_ in the presence/absence of cyt *c*_552_. The obtained results shed light on the potential utilization of immobilized *cd*_1_NiRs in bioelectrochemical devices for biotechnological applications.

## Materials and Methods

### Reagents and proteins

6-amino-1-hexanethiol hydrochloride and 11-amino-1-undecanethiol hydrochloride were purchased from Dojindo; all other chemicals were purchased from Sigma-Aldrich. The reagents were analytical grade and used without further purification. Solutions were prepared with deionized water (18MΩ.cm) from a Millipore MilliQ water purification system. *Mhcd*_1_ (100 μM in 50 mM Tris-HCl buffer, pH 7.6, unless stated otherwise) and cyt *c*_552_ (150 μM in 50 mM Tris-HCl buffer, pH 8) were purified from *M*. *hydrocarbonoclasticus* cells as previously described [26,27].

### Electrode modification and protein immobilization

The nanostructured silver ring electrodes were prepared as previously described [28]. The roughened electrodes were subsequently coated with bifunctional alkanethiol-based SAMs by immersion into 1 mM ethanolic solution of the monolayer. The following pure and mixed SAMs were used: ethanethiol, 1-propanethiol, 1-hexanethiol, 1-undecanethiol, 2-mercaptoethylamine hydrochloride, 6-amino-1-hexanethiol hydrochloride, 11-amino-1-undecanethiol hydrochloride, 6-mercaptohexanoic acid, 11-mercaptoundecanoic acid, 6-mercapto-1-hexanol and 11-mercapto-1-undecanol. The proteins were adsorbed on the SAM-coated electrodes following two different procedures: the modified electrode was i) immersed for 1 hour in a 0.1 μM protein solution (in supporting electrolyte, 12.5 mM potassium phosphate, 12.5 mM K_2_SO_4_, pH 7), then removed and rinsed with supporting electrolyte to eliminate unbound or loosely bound protein or ii) directly placed into the SERR spectroelectrochemical cell containing the supporting electrolyte and 0.1 μM protein, which was allowed to adsorb at open circuit for 30 min; additionally, positive or negative potentials were applied to the electrode during “in-cell” adsorption. The protein-containing solution was afterwards replaced by a protein-free supporting electrolyte. The duration of the immobilization procedure, protein concentration, pH of the buffer and temperature were previously optimized.

### SERR spectroelectrochemistry

The potential-controlled SERR experiments were performed using a home-built spectroelectrochemical cell equipped with an Ag/AgCl (3 M, KCl) reference electrode (210 mV vs. the standard hydrogen electrode, SHE) and a platinum wire counter electrode. The experiments were carried out in 12.5 mM potassium phosphate and 12.5 mM K_2_SO_4_, pH 7, except for nitrite activity assays, where 50 mM MES buffer with 50 mM KCl, pH 6.3 was used. A confocal microscope, equipped with an Olympus 20X objective (working distance of 21 mm, numeric aperture of 0.35), was used for laser focusing onto the sample and light collection in the backscattering geometry. The microscope was coupled to a Raman spectrometer (Jobin Yvon U1000), equipped with a 1200 lines/mm grating and a liquid nitrogen-cooled CCD detector. The 413 nm line from a krypton ion laser (Coherent Innova 302) was used as the excitation source. The laser beam was focused onto the surface of the enzyme modified electrode with a power of ca. 1.5 − 2.5 mW; spectral accumulation time was typically 30 s; 3 − 5 spectra were co-added in each measurement to improve signal to noise (S/N) ratio. The working electrode was kept under constant rotation (600 rpm). The electrode potentials were controlled using a Princeton Applied Research 263A potentiostat. All spectra were subjected to polynomial baseline subtraction; the positions and widths of Raman bands were determined by component analysis as described previously [29]. Redox parameters of *Mhcd*_1_ were obtained by fitting the normalized intensity of the ν_4_ band of the measured, potential dependent SERR spectra to the Nernst equation; in the case of cyt *c*_552_ the SERR spectra were subjected to a component analysis taking into account the ν_4_, ν_3_, ν_2_ and ν_10_ modes, the redox parameters were then estimated from fits of the relative concentrations of the oxidized and reduced species plotted as a function of the electrode potential [29].

Cyclic voltammetry experiments were performed in the SERR spectroelectrochemical cell. The supporting electrolyte solution was thoroughly deoxygenated using oxygen-free argon prior to electrochemical measurements. To evaluate the response of the bioelectrode constructs to nitrite, small volumes of sodium nitrite solutions were successively added to the cell.

All potentials are quoted versus SHE.

### RR spectroscopy

The RR spectra were measured with 413 nm excitation (*vide supra*) in a rotating cuvette (Hellma) filled with ca. 80 μL of sample. Protein concentration was 100 and 150 μM for *Mhcd*_1_ and cyt *c*_552_, respectively. The laser power and accumulation time were 1.5 − 3.5 mW and 20 − 40 s; typically 3 − 10 spectra were co-added in each measurement to improve S/N. The spectra were submitted to component analysis as described in the previous section.

RR potentiometric titrations: The *Mhcd*_1_ samples were prepared in a N_2_ atmosphere, inside an anaerobic chamber (O_2_ < 2 ppm). Step-wise reduction of the ferric enzyme was achieved by addition of small volumes of sodium dithionite solution (1 mM, Tris-HCl 100 mM, pH 7.6); at each point the solution potential was measured with a combined platinum—Ag/AgCl electrode (207 mV vs. SHE). Upon each addition of the reductant, the RR cell was removed from the glove box and the spectra were measured; a fresh aliquot of protein was used for each measurement (i.e. each data point in the titration curve). The protein concentration was 60 μM in a Tris-HCl 50 mM, pH 7.6 buffer containing a mixture of redox mediators at 10 μM each: 1,2-naphthoquinone-4-sulfonic acid (215 mV), 1,2-naphthoquinone (180 mV), trimethylhydroquinone (115 mV), phenazine methosulfonate (80 mV), methylene blue (11 mV), resorufin (−51 mV), indigo disulfonate, (−125 mV), 2-hydroxy-1,4-naphthoquinone (−145 mV), anthraquinone-2-sulfonic acid (−182 mV) and phenosafranine (−255 mV). Redox parameters were obtained as described in previous sections.

Binding of NO to *Mhcd*_1_: Protein concentration was 75 μM in 50 mM Tris-HCl, pH 7.6. The solutions of either resting ferric state, or ferrous *Mhcd*_1_, were first degassed with argon (reduction of the ferric enzyme was achieved by addition of sodium dithionite or sodium ascorbate). The *Mhcd*_1_-NO adducts were prepared inside an anaerobic chamber by the addition of a NO releasing compound (diethylamine NONOate in 10 mM NaOH) to the cell completely filled with the protein solution (10 times the protein concentration). At the pH at which the experiments were performed, each diethylamine NONOate molecule releases 1.5 molecules of NO (t½ = 16 min, at 25°C, pH 7.4). The RR cell was then tightly closed with thick Teflon stoppers which do not allow oxygen diffusion into the cell during RR measurements. The spectra of the ferric and ferrous protein samples were also acquired immediately prior to the addition of diethylamine NONOate.

### UV-Vis spectroscopy

UV-Vis absorption spectra were recorded using a Shimadzu UV-1203 spectrophotometer. The samples were prepared in quartz cuvettes with a path length of 10 mm, sealed with silicone septa. All measurements were performed at room temperature. Protein concentration was 5 μM in Tris-HCl 50 mM, pH 7.6 buffer. The ferric and ferrous NO adducts were prepared as described in the previous section. All measurements were performed inside an anaerobic chamber (O_2_ < 2 ppm).

## Results and Discussion

Our earlier findings revealed that the catalysis of *Mhcd*_1_ depends on the presence of its physiological redox partner, cyt *c*_552_ [23,24]. We have therefore focused on the formation and characterization of a functional catalytic complex between *Mhcd*_1_ and cyt *c*_552_.

### Co-immobilization of cyt *c*_552_ and *Mhcd*_1_

**Fig 1:**
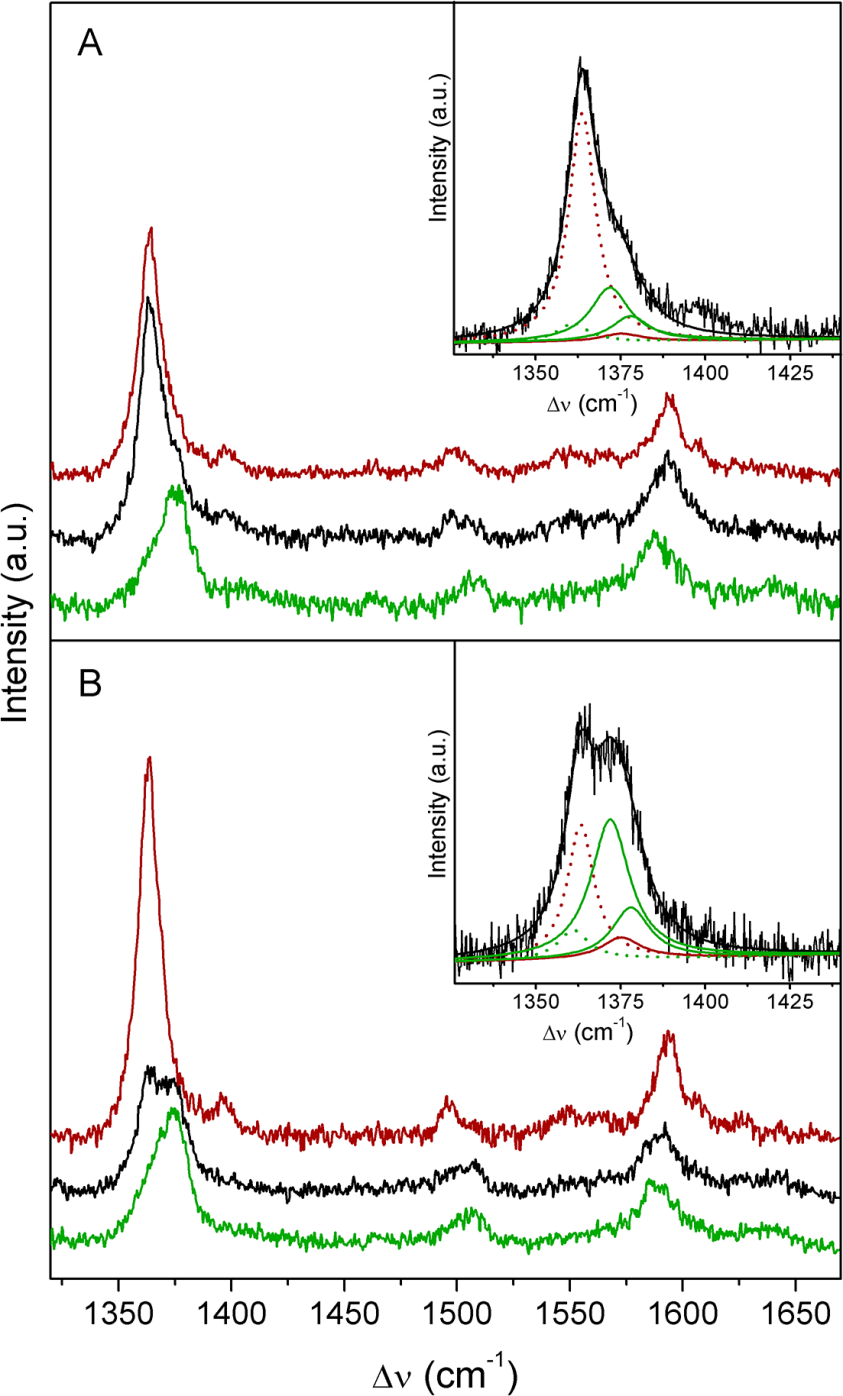
SERR spectra of co-immobilized *Mhcd*_1_ and cyt *c*_552_. **A)** Ag // 6-mercapto-1-hexanol/1-hexanethiol **//** cyt *c*_**552**_ // *Mhcd*_**1**_ and **B)** Ag // 11-amino-1-undecanethiol hydrochloride/1-undecanethiol **//** *Mhcd*_**1**_ // cyt *c*_**552**_ constructs at different poised potentials: 300 mV (green), 200 mV (black) and 0 mV (red). **Inset:** component analysis of experimental spectra (black traces) in ν_**4**_ region of co-adsorbed *Mhcd*_**1**_ and cyt *c*_**552**_ measured at 200 mV; cyt *c*_**552**_ (red) and *Mhcd*_**1**_ (green) populations; overall fit (black). Solid traces designate ferric and dotted traces ferrous ν_**4**_ components. The spectra were recorded with 413 nm excitation; laser power and accumulation time were 1.5 mW and 30 s, respectively.

**Fig 2:**
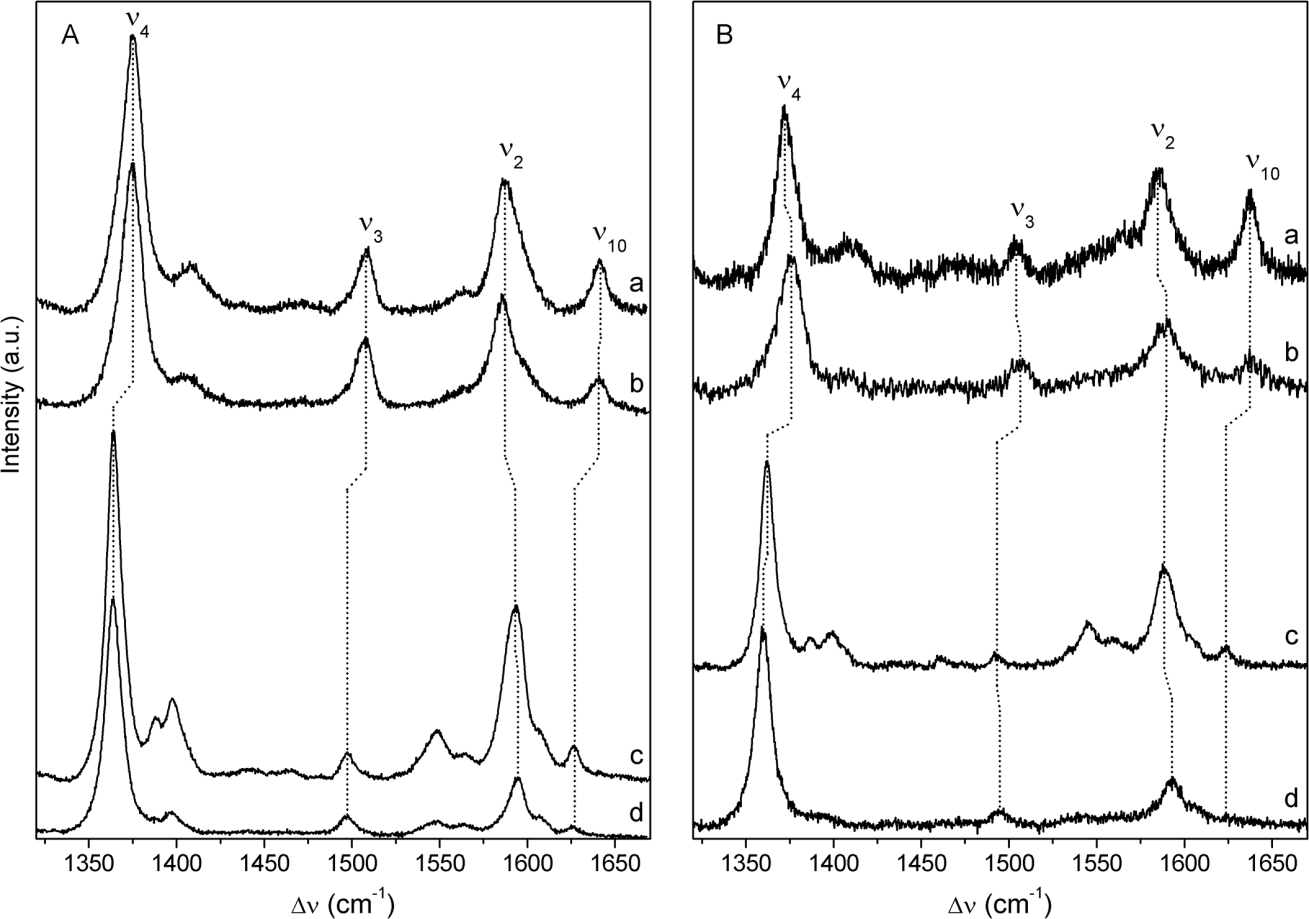
RR and SERR spectra of cyt *c*_552_ and *Mhcd*_1_. **A)** cyt *c*_**552**_: RR spectra of (a) ferric and (c) sodium ascorbate reduced, ferrous protein; SERR spectra of cyt *c*_**552**_ immobilized on 6-mercapto-1-hexanol/1-hexanethiol SAM at electrode potentials of (b) 400 mV and (d) −100 mV. **B)** *Mhcd*_**1**_: RR spectra of (a) ferric and (c) sodium ascorbate reduced, ferrous enzyme; SERR spectra of *Mhcd*_**1**_ on 11-amino-1-undecanethiol hydrochloride/1-undecanethiol SAM at electrode potentials of (b) 300 mV and (d) −300 mV. The spectra were recorded with 413 nm excitation; laser power and accumulation time were 2 − 3 mW and 40 s (RR) or 1.5 − 2.5 mW and 30 s (SERR).

Co-immobilization of cyt *c*_552_ and *Mhcd*_1_ was achieved by sequential incubation of SAM-coated silver electrodes with the two proteins, cyt *c*_552_ followed by *cd*_1_NiR and vice versa, resulting in the following constructs: Ag // SAM // cyt *c*_552_ // *Mhcd*_1_ and Ag // SAM // *Mhcd*_1_ // cyt *c*_552_. For each case, several alkanethiol-based SAMs (e.g. NH_2_^+^-, CH_3_-, COO^-^-and OH-terminated) and their mixtures in different molar ratios were tested. The SERR signal intensities of cyt *c*_552_ were optimized on surfaces coated with 6-mercapto-1-hexanol/1-hexanethiol (OH/CH_3_) and of *Mhcd*_1_ on 11-amino-1-undecanethiol hydrochloride/1-undecanethiol (NH_2_^+^/CH_3_) in 1:1 molar ratios; the former confers mostly polar-hydrophobic surface for cyt *c*_552_ adsorption, and the latter positively charged-hydrophobic surface for *Mhcd*_1_ attachment. These SAMs provided ca. 2 to 3 times stronger signals of cyt *c*_552_ and *Mhcd*_1_, respectively, than the other tested monolayers. Successful co-immobilization of the two proteins on the same electrode construct was demonstrated by SERR (Fig 1), based on individual spectroscopic fingerprints of the ferric and ferrous cyt *c*_552_ and *Mhcd*_1_, which could be identified in the SERR spectra of the complex. Spectral parameters, e.g. band frequencies and widths (Table 1), which were used to deconvolute the SERR spectra of the cyt *c*_552_/*Mhcd*_1_ complexes were derived from RR spectra (Fig 2, traces a and c).

**Table 1:** Frequencies of RR and SERR marker bands of *Mhcd*_1_ and cyt *c*_552_.

	ν_i_ (Δν)/ cm^-1^	ν_4_	ν_3_	ν_2_	ν_10_
**RR**	**Ferric *Mhcd***_**1**_	1372 (11.0); 1378 (13.5)	1505 (11.5)	1585 (14.8)	1637 (10.3)
	**Ferrous *Mhcd***_**1**_	1362 (9)	1494 (8.5)	1589 (14.1)	1623 (9.3)
		^a^ 1362	1494	1582	1624
	**Ferric *Mhcd***_**1**_ **-NO**	^b^ 1370	1503	1587	1635
		^c^ 1377	-	1592	1640
		1363	1492	1582	1624
	**Ferrous *Mhcd***_**1**_ **-NO**	^b^ 1370	1503	1587	1634
		^c^ 1378	-	1593	1640
**SERR**	**Ferric *Mhcd***_**1**_	1372 (13); 1378 (13.5)	1505 (12.8); 1511 (11.9)	1588 (14.4)	1639 (11.5)
	**Ferrous *Mhcd***_**1**_	1361 (10.9)	1493 (10)	1593 (14)	1624 (9)
**RR**	**Ferric cyt *c***_**552**_	1375 (14.1)	1508 (11.5)	1587 (13.4)	1641 (8.6)
	**Ferrous cyt *c***_**552**_	1364 (10.3)	1497 (9.0)	1592 (13.9)	1627 (8.3)
**SERR**	**Ferric cyt *c***_**552**_	1375 (14.1)	1507 (11.5)	1586 (13.4)	1640 (8.6)
	**Ferrous cyt *c***_**552**_	1363 (10.7)	1496 (9.2)	1593 (13.9)	1626 (7.5)

Assignment of marker bands (ν_i_) of *Mhcd*_1_ and cyt *c*_552_ in the ferric and ferrous states determined from the RR and SERR spectra recorded with 413 nm excitation. SERR spectra were measured from SAM-coated Ag electrodes, with cyt *c*_552_ immobilized on 6-mercapto-1-hexanol/1-hexanethiol; ferric and ferrous states were obtained at electrode potentials of 400 mV and −100 mV, respectively, and *Mhcd*_1_ immobilized on 11-amino-1-undecanethiol hydrochloride/1-undecanethiol; ferric and ferrous states were obtained at electrode potentials of 300 mV and −300 mV, respectively. For the *Mhcd*_1_-NO adducts the vibrational modes of the three identified populations, auto-reduced *Mhcd*_1_, 6cLS and 5cHS *d*_1_, are presented. The bandwidths (Δν) used for quantitative component analysis are given in parenthesis.

^a^Auto-reduced in ferric protein (*c* ^2+^).

^b^Ferrous 6cLS NO adduct (*c* ^2+^/6cLS *d*_1_^2+^-NO).

^c^Ferrous 5cHS NO adduct (*c* ^2+^/5cHS *d*_1_^2+^-NO).

Characteristic RR marker bands: ν_4_, ν_3_, ν_2_ and ν_10_ of the His/Met coordinated cyt *c*_552_ are found at 1375, 1508, 1587 and 1641 cm^-1^, respectively, for the ferric and at 1364, 1497, 1592 and 1627 cm^-1^, respectively, for the ferrous protein in solution, indicating a six-coordinated low spin heme (6cLS) configuration (Fig 2A, Table 1) [30,31]. The spectra are comparable with those of cytochrome *c*_551_ from *P*. *aeruginosa*, measured with 413 nm excitation, and reveal small but systematic frequency upshifts [32]. A comparison of RR spectra of ferric and ferrous cyt *c*_552_ in solution with SERR spectra of adsorbed cyt *c*_552_ at positive (400 mV) and negative (−100 mV) potential indicates that the native protein structure is preserved upon immobilization on OH/CH_3_ SAMs (Fig 2A, Table 1). The absence of band broadening also indicates that the orientation of immobilized cyt *c*_552_ was uniform. Note that, under the experimental conditions used in this work, the positive potentials required to achieve complete cyt *c*_552_ reduction (400 mV) did not cause the oxidation of the Ag electrode.

The RR marker bands of *Mhcd*_1_ (ν_4_, ν_3_, ν_2_ and ν_10_ at 1372 cm^-1^, 1505 cm^-1^, 1585 and 1637 cm^-1^ in the ferric and at 1362, 1494, 1589 and 1623 cm^-1^ in the ferrous state, respectively) suggest the presence of the 6cLS heme state. SERR spectra of the immobilized enzyme measured at the positive (300 mV) and negative (−300 mV) potentials show minor shifts in comparison with those observed in RR spectra (Fig 2B, Table 1). Under equivalent experimental conditions, RR spectra of ferrous *Pacd*_1_ reveal a consistent band upshift (ν_4_, ν_3_ and ν_2_ at 1368 cm^-1^, 1500 cm^-1^ and 1597 cm^-1^) [33]. It is noteworthy that in some cases, we observe a shoulder at 1362 cm^-1^ on the ferric ν_4_ band, indicative of laser induced photo-reduction; this population increases proportionally to the laser power and accumulation time. Its contribution in the spectra was kept as low as possible by using a compromise between a reasonable S/N ratio and minimal photo-reduction.

Albeit small, differences in the frequencies of the marker bands between cyt *c*_552_ and *Mhcd*_1_ allow for a complete separation of their SERR spectral contributions when they are simultaneously present on the electrode (Fig 1A and 1B). The presence of the two proteins becomes especially evident upon applying potentials in the 0 − 200 mV range, at which *Mhcd*_1_ is almost or fully oxidized and cyt *c*_552_ nearly or fully reduced (Fig 1, red and black traces). Component analysis of the ν_4_ mode region measured at 200 mV on Ag // OH/CH_3_ // cyt *c*_552_ // *Mhcd*_1_ (Fig 1A, inset) and on Ag // NH_2_^+^/CH_3_ // *Mhcd*_1_ // cyt *c*_552_ (Fig 1B, inset), further reinforces evidence for the presence of the two proteins on the same electrode construct. At electrode potential of 200 mV, the SERR spectrum of Ag // OH/CH_3_ // cyt *c*_552_ // *Mhcd*_1_ complex is dominated by the features of cyt *c*_552_ (Fig 1A, black trace and inset) due to a greater plasmonic enhancement, resultant of the closer proximity of cyt *c*_552_ to the nanostructured metal. In addition, cyt *c*_552_ is already fully reduced at this potential (ν_4_ at 1364 cm^-1^), at which *Mhcd*_1_ is largely oxidized (ν_4_ at 1372 cm^-1^). Similarly, at 300 mV (Fig 1A, green trace) and 0 mV (Fig 1A, red trace), SERR spectra are dominated by the fully oxidized and reduced cyt *c*_552_, respectively. In Ag // NH_2_^+^/CH_3_ // *Mhcd*_1_ // cyt *c*_552_ constructs, in which *Mhcd*_1_ is adsorbed to the electrode prior to cyt *c*_552_, its contribution in the spectra becomes more evident (Fig 1B). At 200 mV (Fig 1B, black trace and inset), the SERR signal is composed of nearly equal contributions from the two proteins, predominantly ferrous cyt *c*_552_ (Fig 1B, inset, dotted red trace) and ferric *Mhcd*_1_ (Fig 1B, inset, solid green traces). Notably, the SERR signal of the two proteins is sufficiently strong even for thick spacers (e.g. 1-undecanethiol, eleven CH_2_ groups).

Despite a direct spectroscopic evidence that *Mhcd*_1_ and cyt *c*_552_ were simultaneously attached to the electrode surface, none of the tested conditions led to catalytically active *Mhcd*_1_, as no catalytic currents were detected in the presence of nitrite (S1 Fig). In the next approach Ag // NH_2_^+^/CH_3_ // *Mhcd*_1_ + solution cyt *c*_552_ and Ag // NH_2_^+^/CH_3_ // cyt *c*_552_ + solution *Mhcd*_1_ constructs were created, in which one of the proteins was immobilized on the electrode surface and the other was confined in its proximity by securing a dialysis membrane around the electrode body. Thus, the partner protein was free to orientate and dock to the immobilized redox partner. Electrocatalytic activity of *Mhcd*_1_ was not measurable under either of these conditions.

In order to understand the reasons for the lack of catalytic activity of *Mhcd*_1_ co-immobilized with cyt *c*_552_, in the next step we probed the thermodynamic (i.e. redox potential, *E°*´) properties of immobilized cyt *c*_552_ and *Mhcd*_1_ in Ag // OH/CH_3_ // cyt *c*_552_ and Ag // NH_2_^+^/CH_3_ // *Mhcd*_1_ constructs respectively, and compared them with the respective features of the two proteins in solution.

### Cyt *c*_552_ immobilized on biocompatible electrodes

**Fig 3:**
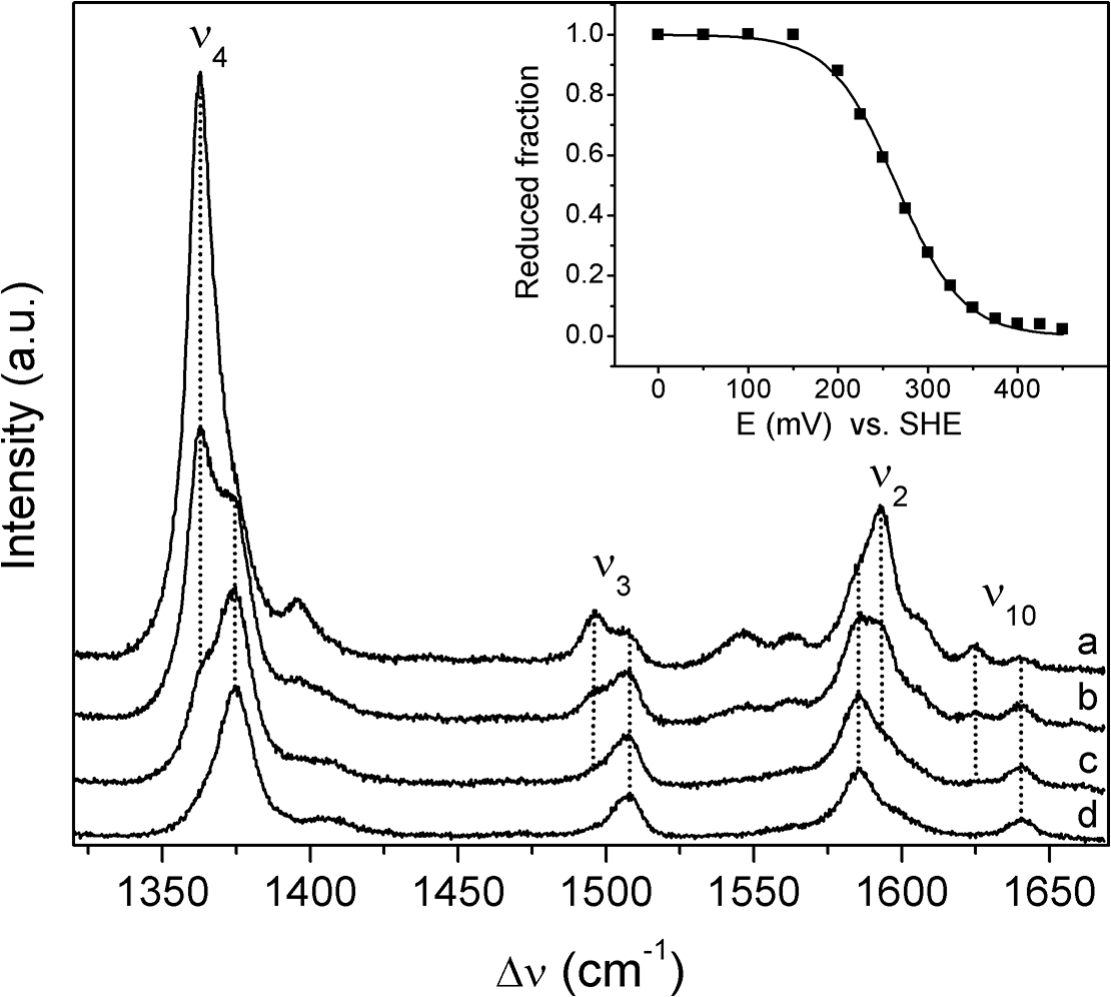
Potentiometric titration of immobilized cyt *c*_552_. SERR spectra of cyt *c*_**552**_ immobilized on 6-mercapto-1-hexanol/1-hexanethiol SAM-coated Ag electrode recorded at electrode potentials of (a) to (d) 250, 300, 350 and 450 mV. All spectra were measured with 413 nm excitation; laser power and accumulation time were 1.5 mW and 30 s, respectively. **Inset:** relative concentration of ferrous protein (squares) plotted as a function of the electrode potential. The solid line represents a fit of the experimental data to the Nernst equation, yielding *E°*´ = 262 ± 5 mV, *z* = 0.70 ± 0.02.

First, the redox potential of the immobilized cyt *c*_552_ was determined by electrochemical redox titrations followed by SERR spectroscopy (Fig 3). The component analysis of the potential dependent SERR spectra of cyt *c*_552_ in which ν_4_, ν_3_, ν_2_ and ν_10_ modes were considered, provides the relative spectral contributions of the ferric and ferrous heme *c*. The spectral parameters (e.g. frequencies and band widths) of each vibrational mode, defined in RR spectra of ferric and ferrous protein, were kept constant such that the only variables at a given potential are the amplitudes of the individual component spectra. The apparent redox potential was estimated from fits to the relative concentrations of the oxidized and reduced species plotted as a function of the electrode potential. The Nernst plot (Fig 3, inset) reveals a redox transition at *E°*´ = 262 ± 5 mV and an apparent number of transferred electrons, *z*, of *z* = 0.70 ± 0.02 for the immobilized cyt *c*_552_, which is in good agreement with UV-Vis potentiometric titrations of the protein in solution (~ 260 mV) [30]. Additionally, the SERR electrode constructs (Ag // OH/CH_3_ // cyt *c*_552_) were characterized by cyclic voltammetry (S2 Fig); cyt *c*_552_ displayed one-electron quasi-reversible electrochemistry, with peak separations up to 50 mV in the studied scan rate range (0.01 to 0.5 V s^-1^) and widths at half height around 130 mV. The peak currents varied linearly with the scan rate in the whole range tested, thus demonstrating that cyt *c*_552_ was adsorbed on the surface of the modified electrode. The *E°*´ (249 ± 2 mV) was determined by the average of anodic and cathodic peak potentials; it was independent of the scan rate and comparable with the value derived from the spectroscopic experiments. Taken together, these results demonstrate that cyt *c*_552_ retains its redox properties and native structure (*vide supra*) upon immobilization on OH/CH_3_ SAM-coated silver electrodes.

### *Mhcd*_1_ immobilized on biocompatible electrodes

The analysis of the redox behavior of the immobilized *Mhcd*_1_ is not as straightforward, due to i) possible ambiguities related to the presence of two hemes and ii) the presence of more than one spin species, particularly evident upon component analysis of the RR and SERR spectra of the ferric protein (cf. Table 1). Although the high frequency region of the RR spectra of the resting state *Mhcd*_1_ (Fig 2B), obtained with 413 nm excitation, reveals features characteristic of *c*-type heme proteins, in principle, contributions from both hemes, *c* and *d*_1_, could be expected since their respective Soret bands coincide in the ferric enzyme (S3 Fig, trace a). However, a comparison of the extinction coefficients for the ferric isolated *d*_1_ cofactor (30.5 mM^-1^.cm^-1^) and His/Met (105 mM^-1^.cm^-1^) or His/His (97.5 mM^-1^.cm^-1^) coordinated *c* hemes [34,35], found in *cd*_1_NiRs from *P*. *aeruginosa* (*Pacd*_1_) and *P*. *pantotrophus* (*Ppcd*_1_), respectively, indicates that the resonance enhancement and therefore the spectral contribution of heme *d*_1_ should be considerably lower than that of heme *c*. In the reduced enzyme, heme *c* is selectively enhanced with 413 nm excitation [33] since the low intensity Soret band of the ferrous heme *d*_1_ is further red-shifted to 460 nm (S3 Fig, trace d). Therefore, we attribute spectra of ferric and ferrous enzyme measured with 413 nm to heme *c* of *Mhcd*_1_.

**Fig 4:**
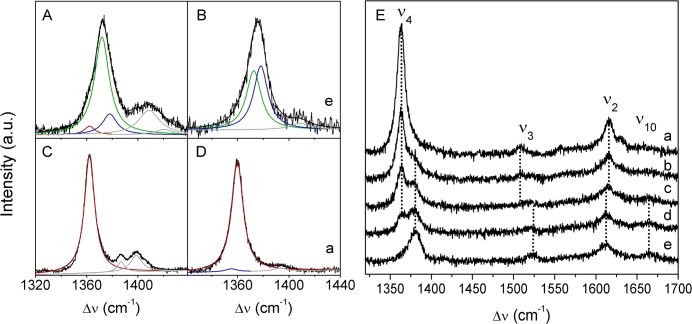
RR and SERR spectra of *Mhcd*_1_. Component analysis of the ν_**4**_ region of: RR spectra of **A)** ferric and **C)** ferrous *Mhcd*_**1**_ and SERR spectra of *Mhcd*_**1**_ immobilized on a 11-amino-1-undecanethiol hydrochloride/1-undecanethiol SAM at electrode potentials of **B)** 300 mV and **D)** −300 mV; green and red solid lines represent native ferric and ferrous populations, respectively, blue line accounts for non-native populations; gray line for non-assigned bands and black line for the overall fit. Red line in panel A indicates traces of photo-reduced protein. **E)** SERR spectra of *Mhcd*_**1**_ recorded as a function of the electrode potential, from (a) to (e) −150, −50, 50, 100 and 300 mV. The spectra were recorded with 413 nm excitation; laser power and accumulation time were 3 mW and 40 s (RR) or 2.5 mW and 30 s (SERR), respectively.

Deconvolution of SERR spectra of the oxidized *Mhcd*_1_ immobilized on NH_2_^+^/CH_3_ SAMs and measured at potentials ≥ 200 mV, reveals that the ν_4_ mode is composed of two ferric components, centered at 1372 cm^-1^ and 1378 cm^-1^. Likewise, the component analysis of the RR spectra of *Mhcd*_1_ in the resting state indicates that in the majority of cases the ν_4_ mode has an additional component at 1378 cm^-1^, which depending on the protein fraction, accounts for up to 15% of the ν_4_ intensity (Fig 4A and 4B, blue trace; Table 1). We attribute the 1372 cm^-1^ band to the native form, as it is largely dominant in solution, and the 1378 cm^-1^ component to a non-native population and designate them as ox_1_ and ox_2_, respectively. In the spectra of immobilized *Mhcd*_1_ (Fig 4B), we observe approx. 1:1 mixture of native and non-native populations. Both ox_1_ and ox_2_ are in 6cLS state, as revealed by the frequency of the broadened ν_3_ mode (Δν_3_ (SERR) = 17 cm^-1^, Δν_3_ (RR) = 11 cm^-1^, Table 1), possibly representing two populations carrying different axial ligands. The SERR spectrum of the enzyme recorded at negative potential (≤ −300 mV, Fig 4D) is indicative of the native ferrous enzyme (red_1_) identified in the RR spectra of the ascorbate-reduced enzyme in solution (Fig 4C). After prolonged exposure of the enzyme to negative potentials and laser beam, a second non-native component of ferrous *Mhcd*_1_ (ν_4_ at 1355 cm^-1^) could be identified in SERR spectra (Fig 4D). Note a slight broadening (1 − 2 cm^-1^) of some SERR bands relative to those observed in RR spectra (Table 1), which is most likely related to orientation heterogeneity of the immobilized *Mhcd*_1_ molecules.

**Fig 5:**
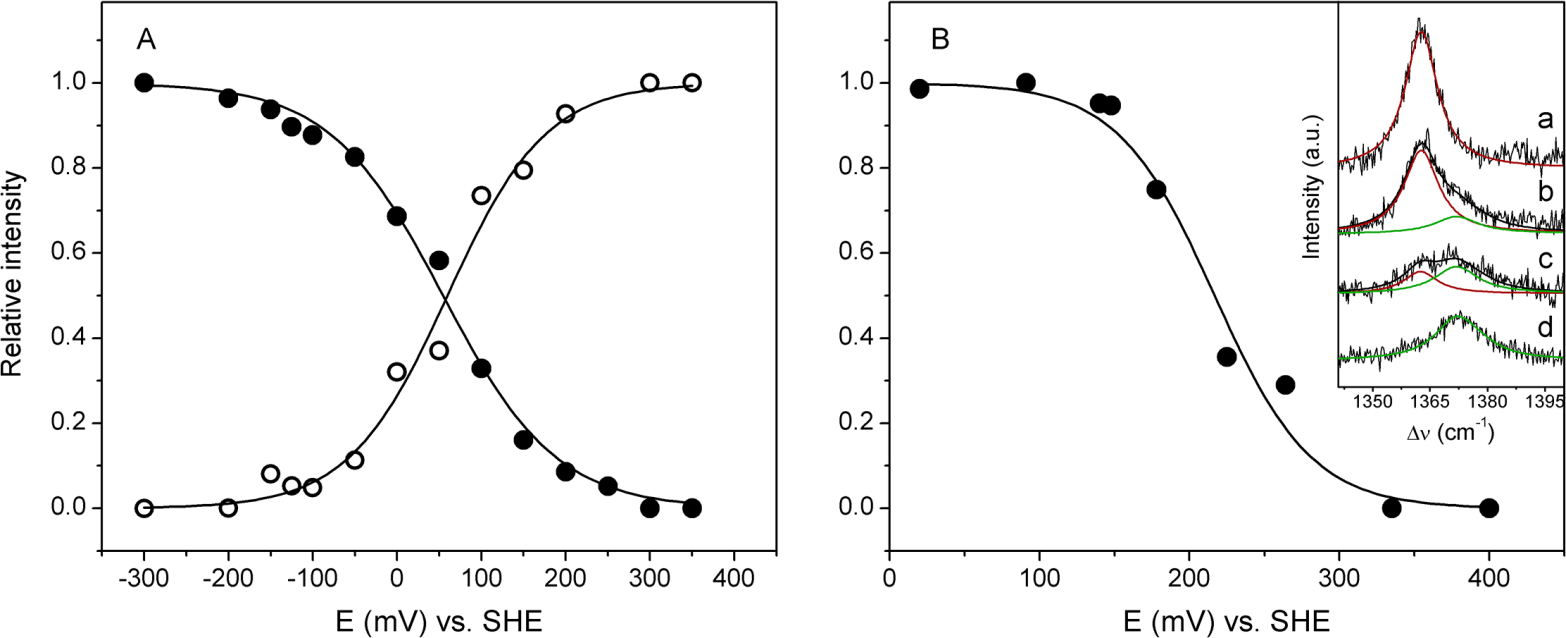
Redox titrations of *Mhcd*_1_ in immobilized and solution state. **A)** SERR spectroelectrochemical titration of *Mhcd*_**1**_ adsorbed on 11-amino-1-undecanethiol hydrochloride/1-undecanethiol coated electrodes. Data points correspond to the relative intensities of ferrous (ν_**4**_ at 1362 cm^-1^; solid circles) and ferric (ν_**4**_ at 1372 cm^-1^; open circles) heme *c* populations, as a function of the electrode potential. Solid lines represent fits of the Nernst equation, *E°*´ ~ 70 mV, *z* = 0.44, to the experimental data points. **B)** RR redox titration of *Mhcd*_**1**_ in solution. The relative intensities of the reduced population are represented as a function of the solution potential, solid circles. The Nernst equation was fitted to the data (black line) with *E°*´ = 220 ± 5 mV, *z* = 0.90. **Inset:** ν_**4**_ band of RR spectra measured at solution potentials of (a) 90, (b) 180, (c) 225 and (d) 335 mV. Component spectra represent ferrous (red) and ferric (green) ν_**4**_ populations and overall fit (black). The spectra were recorded with 413 nm excitation, with 2 − 3 mW laser power and 40 s accumulation time. **Note:** Sample preparation for solution RR titrations was performed in anaerobic conditions (glove box). Upon each addition of the reductant, the RR cell was removed from the glove box and the spectra were measured; a fresh aliquot of protein was used for each data point.

In the next step, we probed the redox behavior of *Mhcd*_1_ attached to NH_2_^+^/CH_3_ coated electrodes employing potential-dependent SERR spectroscopy. The SERR spectra, measured in the potential range between −400 and 350 mV (Fig 4E), were subjected to a component analysis, as described for cyt *c*_552_ (*vide supra*). In the case of *Mhcd*_1_ only ν_4_ modes were considered, due to a poorer quality of the spectra. At each electrode potential, the spectra could be consistently analyzed on the basis of the two ferric (ox_1_ and ox_2_) and one ferrous (red_1_) components. Also, relative intensities (I_i_) were used in the analysis instead of relative concentrations (c_i_), c_i_ = f_i_ I_i_; this is a frequently adopted strategy when the factors f_i_, that are proportional to the relative reciprocal SERR cross sections of the species *i*, are unknown [20]. The apparent redox potentials were estimated from fits to the relative band intensities plotted as a function of the electrode potential (Fig 5A). The Nernst plots reveal a redox transition at *E°*´ ~ 70 mV for the native redox couple (ox_1_/red_1_) of the immobilized *Mhcd*_1_, which is assigned to heme *c*. The non-native population, as estimated from the potential-dependent contributions of ox_2_, shows a much broader transition at *E°*´ ~ 60 mV.

In parallel, the reduction potential of *Mhcd*_1_ was determined in solution by potentiometric titrations of the enzyme monitored by RR spectroscopy (Fig 5B). The spectra were recorded after a stepwise addition of sodium dithionite to the buffer containing the oxidized enzyme and a cocktail of redox mediators. The relative amounts of the reduced and oxidized populations were determined from the component analysis of ν_4_ (Fig 5B, inset) at each solution potential. As described for the analysis of the SERR spectra, the ν_4_ was fitted with two oxidized (ox_1_ and ox_2_) and one reduced (red_1_) components. The relative amount of red_1_ species plotted against the solution potential, shows a redox transition at *E°′* = 220 ± 5 mV and *z* = 0.9 ± 0.1. The determined redox potential is in agreement with previous data from UV-Vis titrations of *Mhcd*_1_ which suggested *E°′* (*c*) = 234 mV (and *E°′* (*d*_1_) = 200 mV) [26]. Besides, this value is comparable with the redox potential of *Pacd*_1_ (*E°′* (*c*, *d*_1_) ~ 280 mV) and to that of *Ppcd*_1_ semi-apoprotein (*E°′* (*c*) = 242 mV) [36,37].

A comparison of SERR and RR titrations shows that the redox transition in immobilized *Mhcd*_1_ is ~150 mV lower than the value determined from the solution studies. Also, the number of transferred electrons in SERR titrations was consistently below 0.5, indicating that the electronic coupling of the protein’s redox centers with the metal surface was not efficient. We associate the redox potential shift of the “native” portion of heme *c* in *Mhcd*_1_ with immobilization induced conformational changes of the secondary structure that can significantly alter hydrophobicity of the heme pocket. While we can exclude major structural perturbations that influence the spin and coordination state of the heme *c*, they cannot be ruled out in the case of heme *d*_1_, which could not be individually probed in the present work. According to Kassner’s relation, opening of the cavity of heme *c* to the solvent and an increased polarity of the heme environment can result in a downshift of redox potentials of up to 0.2 V [38], which is consistent with the shift observed here. *cd*_1_NiRs are actually prone to structural alterations, as a part of catalytic activation. For example, as demonstrated by X-ray crystallography, redox linked conformational changes in *Ppcd*_1_ lead to a loss of hydrophobic interactions and hydrogen bond breakage between the domains harboring hemes *c* and *d*_1_, resulting in increased water exposure of the interface between the two domains [12].

**Fig 6:**
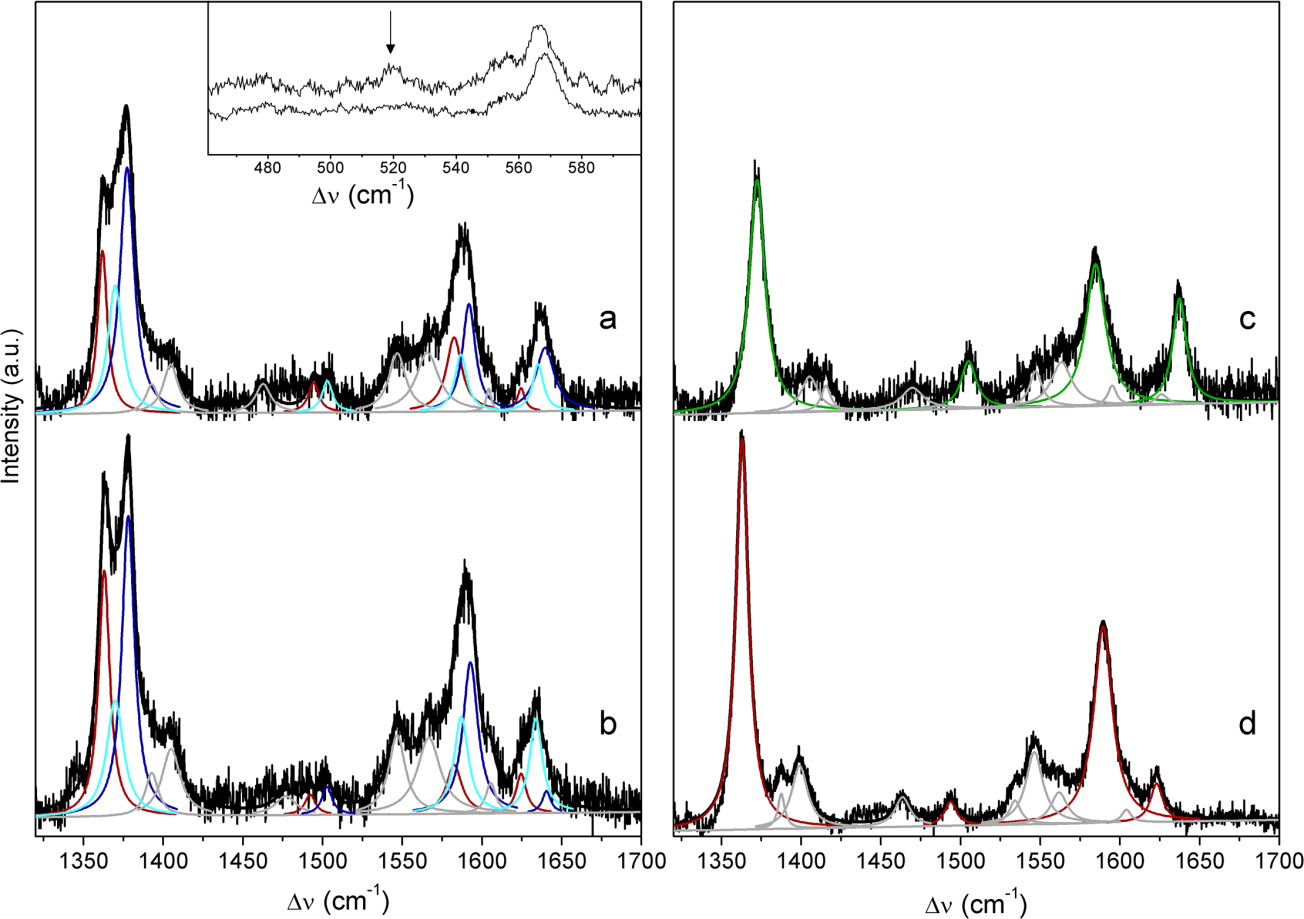
RR spectra of *Mhcd*_1_-NO adducts and *Mhcd*_1_ prior to NO binding. (a) Ferric and (b) ferrous NO adducts of *Mhcd*_**1**_ measured upon addition of diethylamine NONOate; (c) ferric and (d) ferrous *Mhcd*_**1**_ prior to addition of NO. The spectra were measured with 413 nm excitation, 1.6 mW laser power and 40 s accumulation time. Component spectra represent ferrous population (red), 6cLS NO adduct (light blue), 5cHS NO adduct (blue), ferric population (green), non-assigned bands (gray) and overall fit (black). **Inset:** low frequency region of RR spectra of ferric *Mhcd*_**1**_-NO adduct (top trace) and ferric *Mhcd*_**1**_ (bottom trace). The arrow designates the (*d*_**1**_)Fe-NO stretching coordinate of 5c nitrosyl *Mhcd*_**1**_ complex.

We further tested if the immobilized *Mhcd*_1_ was capable of NO binding. The UV-Vis spectra of ferric and ferrous *Mhcd*_1_, recorded upon addition of NO-releasing diethylamine NONOate, indicate that NO binds to the heme *d*_1_ of both forms, as judged by the spectral changes, e.g. Soret band intensity increase, suggesting a blue shift of the 460 nm band of the ferrous *d*_1_ (S3 Fig, trace c) and appearance of an additional band at 644 nm, characteristic of the NO bound 6sLS *d*_1_ heme (S3 Fig, inset traces b and c). Similarly, the RR spectra of both ferric and ferrous *Mhcd*_1_-NO adducts in solution are indicative of several co-existing spin configurations (Fig 6). Due to the blue shift of the Soret band of heme d_1_ upon NO binding, both hemes are probed with 413 nm excitation. Component analysis of the ferric-NO adduct (Fig 6, trace a) reveals a species with ν_4_, ν_3_, ν_2_, and ν_10_ at 1362, 1494, 1582 cm^-1^, and 1624 cm^-1^, respectively. We attribute this species to the ferrous heme *c* (Fig 6A, red), which was also observed in the UV-Vis spectra (S3 Fig, trace b). We identified two additional species, a minor contribution with the corresponding marker bands at 1370, 1503, 1587, and 1635 cm^-1^ (Fig 6A, light blue), and the prevailing species, with the marker bands ν_4_, ν_2_, and ν_10_ at 1377, 1592, and 1640 cm^-1^ (Fig 6A, blue), respectively (cf. Table 1). These two species were not observed in the spectra of ferric (or ferrous) enzyme measured prior to addition of the NO donor (Fig 6, traces c and d). In analogy to other NO binding heme proteins, we attribute the minority species to the ferrous 6cLS NO adduct (i.e. *c* ^2+^/6cLS *d*_1_^2+^-NO), and the second species to the ferrous five-coordinated high spin (5cHS) NO adduct in which the proximal ligand of heme *d*_1_ has been detached (i.e. *c* ^2+^/5cHS *d*_1_^2+^-NO) [39–41]. Nitric oxide is well-known as a strong trans-destabilizing ligand that in its heme complexes can cause disruption of the bond between the heme iron and proximal His ligand, resulting in 5cHS Fe^2+^-NO complex. The atypically high frequencies of the marker bands of NO adducts, also observed in model compounds and various NO binding heme proteins, have been attributed to a decrease in the electron density of the π* antibonding orbitals of the porphyrin macrocycle by back-bonding to NO through the iron d_π_ orbitals [42,43]. Further evidence for the formation of a 5c nitrosyl *Mhcd*_1_ complex comes from the low-frequency region (300 − 600 cm^-1^) of the RR spectra, where an additional broad band appears at ~ 520 cm^-1^ in the presence of NO (Fig 6, inset upper trace). Despite of its very low intensity, this band is clearly absent in the spectra of the ferric protein. The frequency of the band falls into the 520 − 526 cm^-1^ range observed for the Fe-NO stretching in 5c-NO adducts of *c*-type cytochromes [39–42]. The 6cLS nitrosyl adducts of heme proteins tend to have a stronger Fe^2+^-NO bond, with the stretching frequency in the range of 536 − 580 cm^-1^ [39,41], but due to a relatively low contribution of this species (Fig 6A, light blue), it could not be detected in the spectra of *Mhcd*_1_. Actually, the 6cLS Fe^2+^-NO stretching coordinate has been detected at very high frequency (~ 585 cm^-1^) in *Pacd*_1_-NO adduct, which has been rationalized in terms of the particular structural characteristics of the heme *d*_1_ in comparison to other hemes [4]. The features of ferrous heme *c*, observed in UV-Vis and RR spectra of ferric *Mhcd*_1_-NO mixture, have been previously reported for *cd*_1_NiR-NO complexes from other organisms and were ascribed to auto-reduction [44–46]. We can hypothesize that cross-ET from heme *d*_1_ to heme *c* can take place. In fact, a similar scenario was proposed for *Ppcd*_1_, in which after nitrite reduction, an internal ET can occur from heme *d*_1_^2+^-NO to heme *c* ^3+^, resulting in ~ 45% of ferrous heme *c* present in solution, implying the formation of an approximately equimolar mixture of *c* ^2+^/*d*_1_^2+^-NO^+^ and *c* ^3+^/*d*_1_^2+^-NO [47]. The component analysis of the ferrous-NO adduct of *Mhcd*_1_ (Fig 6, trace b) reveals the same species described for the ferric complex.

Based on deconvoluted RR spectra of *Mhcd*_1_-NO adducts, we demonstrate here that *Mhcd*_1_ is capable of forming a NO-bound 5cHS state in which the proximal His is detached from the heme. A similar scenario was observed in NO binding and sensing proteins e.g. GSUs, CooA, sGC, etc [39–41], but formation of 5cHS NO adduct was not previously reported in *cd*_1_NiRs. Despite the quite distinct RR fingerprint of *Mhcd*_1_-NO complex, it could not be observed in SERR spectra of immobilized *Mhcd*_1_, indicating that in neither of the tested conditions (*vide infra*) the enzyme was properly oriented for NO binding.

No further insights could be obtained from *Mhcd*_1_ immobilized under different conditions and thus possibly alternative orientations. When *Mhcd*_1_ was attached onto pure hydrophobic, OH/CH_3_- or COO^-^/CH_3_- terminated SAMs, SERR signals were not stable, as exposure to potentials below 200 mV led to a signal loss after approximately 5 minutes. Additionally, the SERR signal intensity was ca. 3 times weaker on these SAMs in comparison with that on NH_2_^+^/CH_3_ coated electrodes (*vide supra*). The protein could not be immobilized on pure hydroxyl-terminated SAMs, regardless of the immobilization conditions (e.g. “in-cell” or immersion of the electrode into concentrated protein solution externally; positive vs. negative electrode potentials vs. open circuit for “in-cell” immobilization; duration of the immobilization procedure or protein concentration; pH of the buffer; temperature; etc.), while the attempts to attach *Mhcd*_1_ on carboxylate-, and pure amino-terminated SAMs resulted in weak or redox inactive signals.

In conclusion, we demonstrated in this work that among diverse alkanethiol-based SAMs which were tested for immobilization of *Mhcd*_1_, only ´diluted´ positively charged surfaces (mixed NH_2_^+^/CH_3_ monolayers) resulted in the attachment of a stable, redox active enzyme. The apparently structurally intact redox couple, associated with heme *c*, reveals ca. 150 mV negative shift of the redox potential in comparison with the solution value. Cyt *c*_552_, on the other hand, preserves its structural and thermodynamic properties in the immobilized state. Neither adsorbed *Mhcd*_1_ nor cyt *c*_552_/*Mhcd*_1_ complexes assembled under different immobilization conditions were capable of nitrite reduction. Most likely, the altered redox properties, together with an orientation of the immobilized *Mhcd*_1_ that is unfavorable for efficient heterogeneous ET and NO binding, are responsible for the lack of catalytic activity of the immobilized *Mhcd*_1_. Clearly, very specific docking/orientation between *Mhcd*_1_ and its redox partner needs to be achieved, as also demonstrated for e.g. CYPOR and CYP couple, for which both ET and allosteric modulation were found to be highly dependent on the intermolecular interaction [48]. Taken together, the present study reveals that the development of 3^rd^ generation nitrite biosensor based on *Mhcd*_1_ is not feasible at this point. Electrocatalysis of *Mhcd*_1_ critically depends on interactions with its physiological electron donor.

## Supporting Information

S1 Fig Cyclic voltammograms of Au // 6-mercapto-1-hexanol /6-hexanethiol // cyt *c*_552_ // *Mhcd*_1_ constructs. Cyclic voltammograms in the absence of nitrite (black line), and in the presence of 3 mM nitrite (red dashed line). Scan rate 50 mV/s. Supporting electrolyte: MES buffer 50 mM with 50 mM KCl, pH 6.3. The peaks correspond to the reversible electrochemical oxidation/reduction of cyt *c*_552_.
(TIF)

S2 Fig Scan rate dependence of the electrochemical response of cyt *c*_552_ immobilized on 6-mercapto-1-hexanol/6-hexanethiol coated Ag electrodes. **A)** Cyclic voltammograms at varying scan rates (0.01, 0.02, 0.035, 0.05, 0.075, 0.1, 0.2, 0.3 and 0.5 V/s). **B)** Variation of the anodic (squares) and cathodic (circles) peak currents of adsorbed cyt *c*_552_ as a function of the scan rate. Supporting electrolyte: 12.5 mM phosphate buffer and 12.5 mM K_2_SO_4_, pH 7.0.
(TIF)

S3 Fig Electron absorption spectra of *Mhcd*_1_. Ferric enzyme in the (a) absence and (b) presence of diethylamine NONOate and ferrous *Mhcd*_1_ in the (c) presence and (d) absence of the NO donor. *Mhcd*_1_ was 5 M in 50 mM Tris-HCl buffer, pH 7.6; protein was reduced with sodium ascorbate. Asterisk designates the Soret band of reduced heme *d*_1_ (460 nm) and the arrow marks the 644 nm band indicative of NO binding to the ferric and ferrous enzyme.
(TIF)
