# Supplementary figures and images for: SERR Spectroelectrochemical Study of Cytochrome cd 1 Nitrite Reductase Co-Immobilized with Physiological Redox Partner Cytochrome c 552 on Biocompatible Metal Electrodes
Source: PLoS One. 2015 Jun 19;10(6):e0129940. doi: 10.1371/journal.pone.0129940 (PMC4474632; doi:10.1371/journal.pone.0129940)

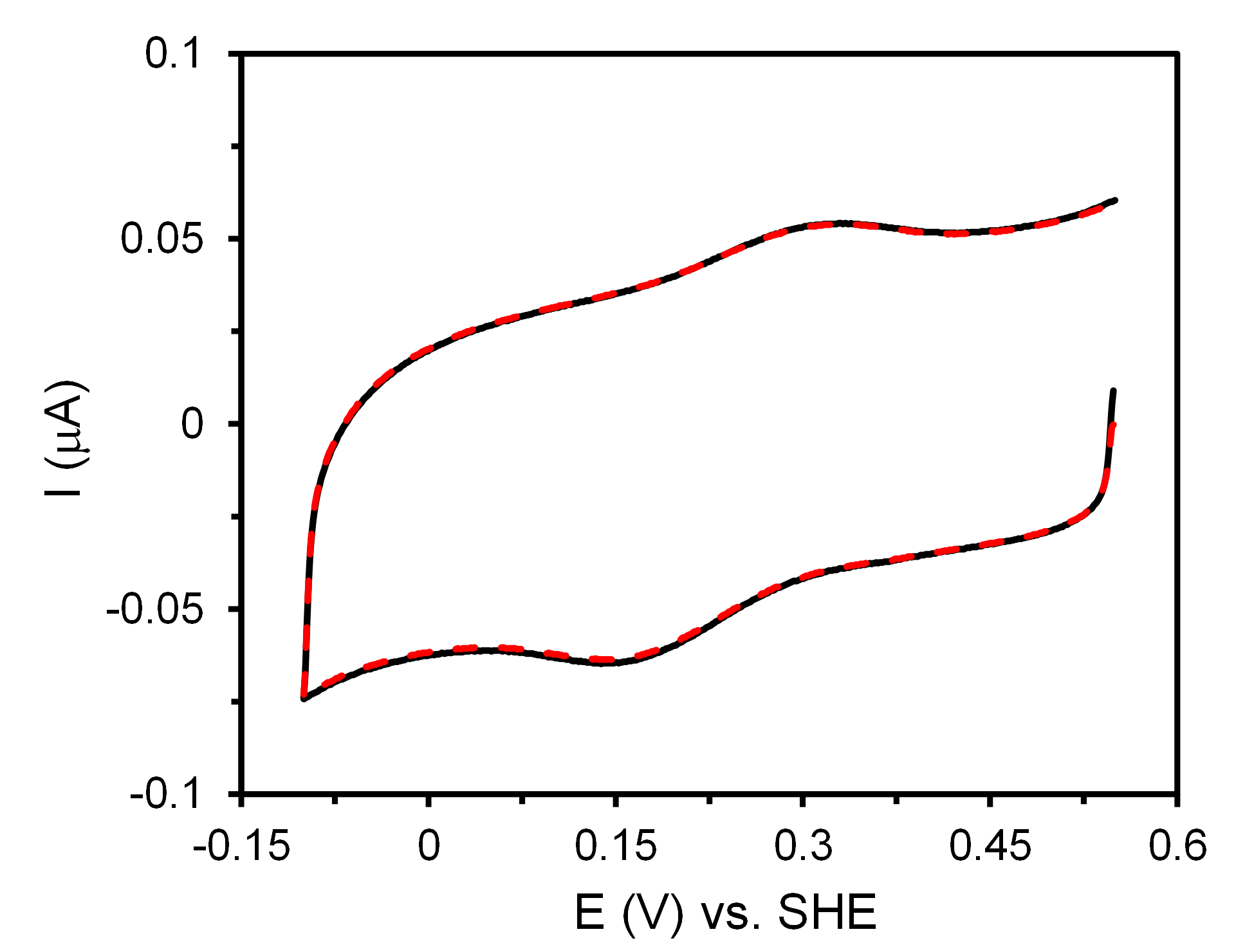

Supplement: S1 Fig — Cyclic voltammograms in the absence of nitrite (black line), and in the presence of 3 mM nitrite (red dashed line). Scan rate 50 mV/s. Supporting electrolyte: MES buffer 50 mM with 50 mM KCl, pH 6.3. The peaks correspond to the reversible electrochemical oxidation/reduction of cyt c 552. (TIF) [file pone.0129940.s001.tif]

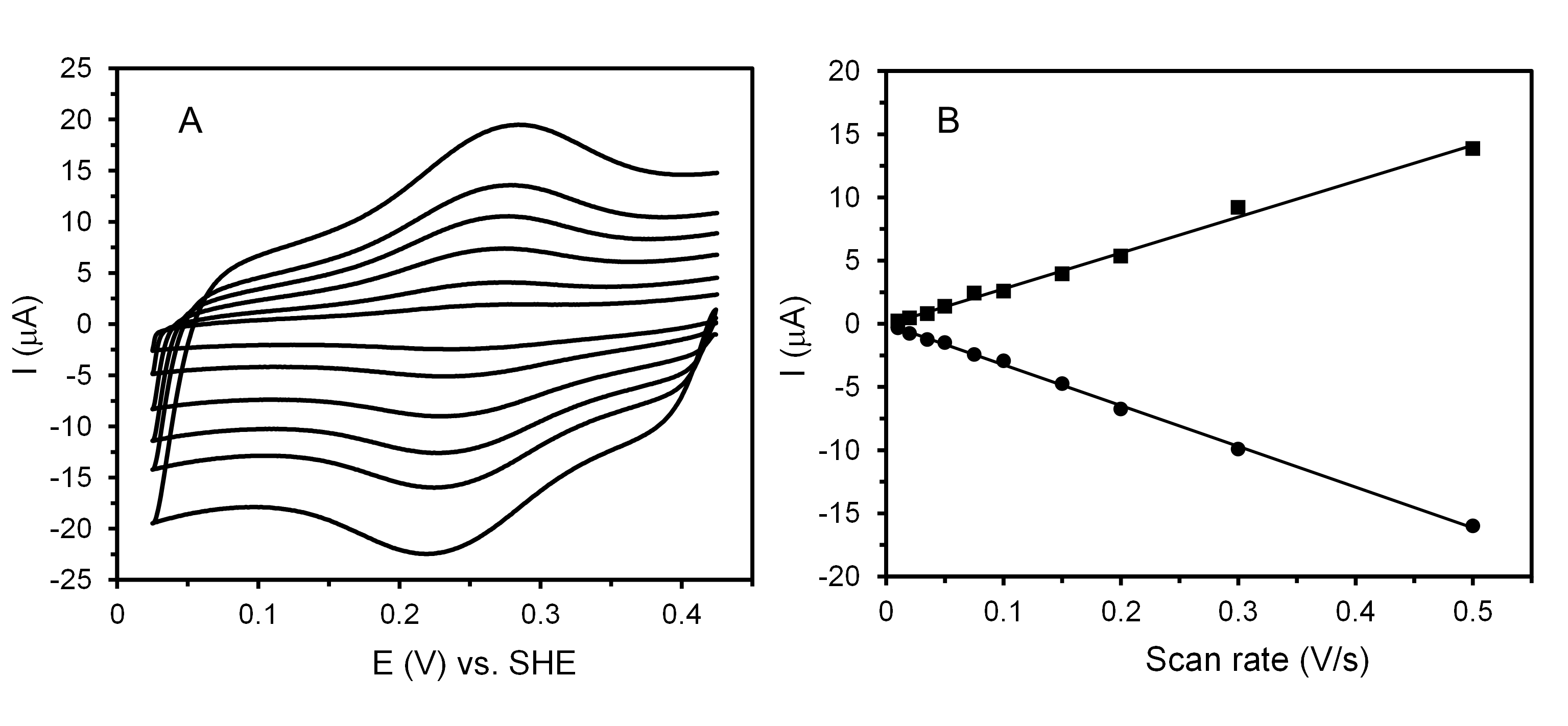

Supplement: S2 Fig — A) Cyclic voltammograms at varying scan rates (0.01, 0.02, 0.035, 0.05, 0.075, 0.1, 0.2, 0.3 and 0.5 V/s). B) Variation of the anodic (squares) and cathodic (circles) peak currents of adsorbed cyt c 552 as a function of the scan rate. Supporting electrolyte: 12.5 mM phosphate buffer and 12.5 mM K2SO4, pH 7.0. (TIF) [file pone.0129940.s002.tif]
